# Supplementary material for: Association between oral microbiome and seven types of cancers in East Asian population: a two-sample Mendelian randomization analysis
Source: Front Mol Biosci. 2023 Nov 21;10:1327893. doi: 10.3389/fmolb.2023.1327893 (PMC10702768; doi:10.3389/fmolb.2023.1327893)
Supplement: Supplementary file 1 [file DataSheet1.ZIP › Supplementary Materials/MR plots of saliva/saliva═╝/colorectal cancer/pheno.1211_to_colorectal cancer_forest.pdf]

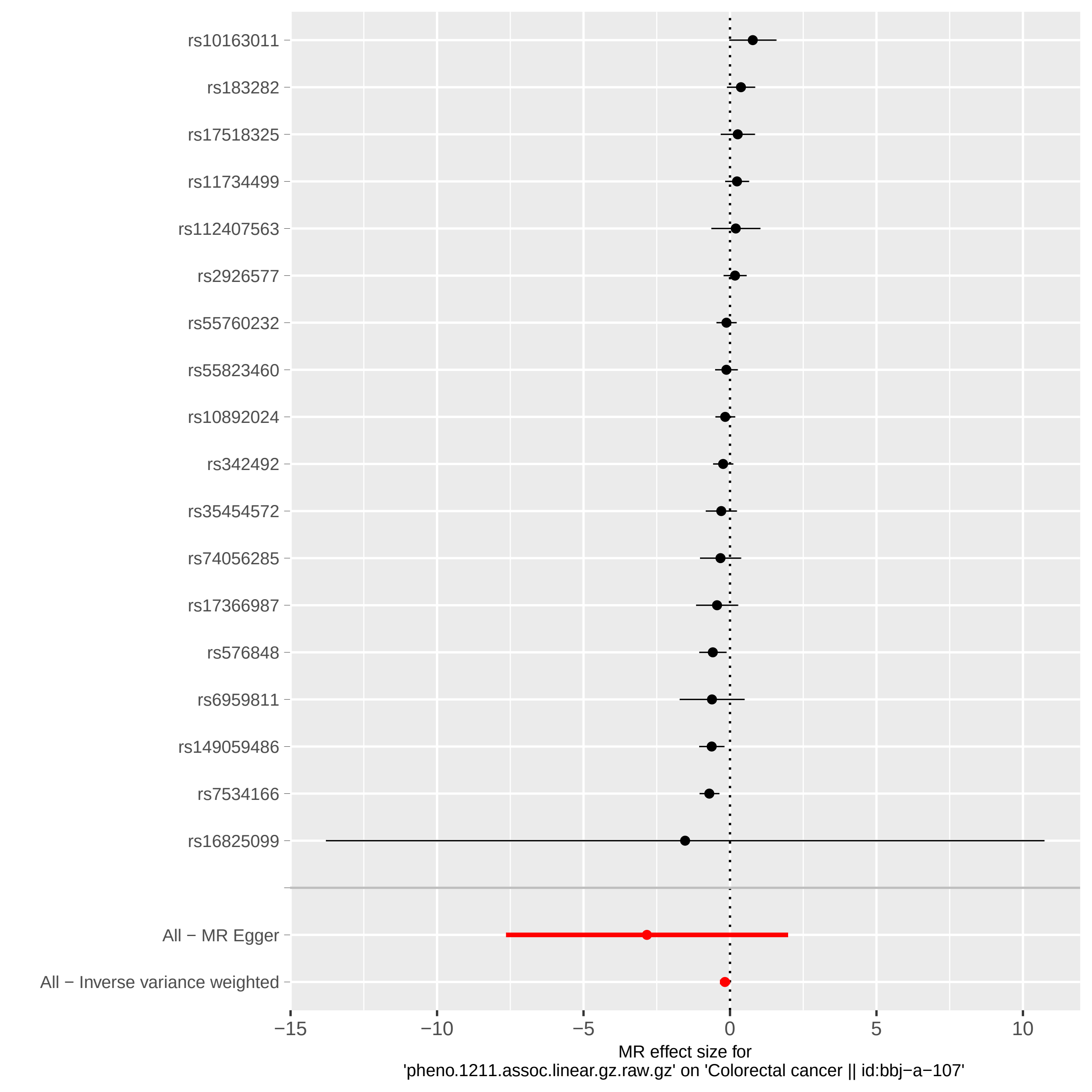
rs10163011
rs183282
rs17518325
rs11734499
rs112407563
rs2926577
rs55760232
rs55823460
rs10892024
rs342492
rs35454572
rs74056285
rs17366987
rs576848
rs6959811
rs149059486
rs7534166
rs16825099
All − MR Egger
All − Inverse variance weighted
−15
−10
−5
0
5
10
MR effect size for
'pheno.1211.assoc.linear.gz.raw.gz' on 'Colorectal cancer || id:bbj−a−107'
